# Memories of Cas Jeekel, friend,  colleague, and role model

**DOI:** 10.3897/zookeys.156.2215

**Published:** 2011-12-20

**Authors:** Richard L. Hoffman

**Affiliations:** 1 Virginia Museum of Natural History, Martinsville, Virginia 24112, USA

Nearly everyone has experienced an event that exerted a profound influence on some aspect of their life; often the full impact of that milestone is not fully appreciated until much later through the filter of retrospection. The first such event that established the course of my own scientific career occurred in November of 1946, when, searching for some group of animals to investigate, I attempted to identify some julid millipeds and discovered that the only comprehensive resource on North American species was published in 1893. The second came in the form of a letter dated 20 August 1949 from a certain C. A.W. Jeekel at the Amsterdam Zoological Museum, introducing himself as a beginning student of myriapods, and inquiring about the availability of certain type specimens in the U.S. National Museum. Thus began an exchange of ideas and information that evolved into a personal friendship that endured for more than 60 years, and resulted in reshaping my approach to taxonomy.

**Figure:**
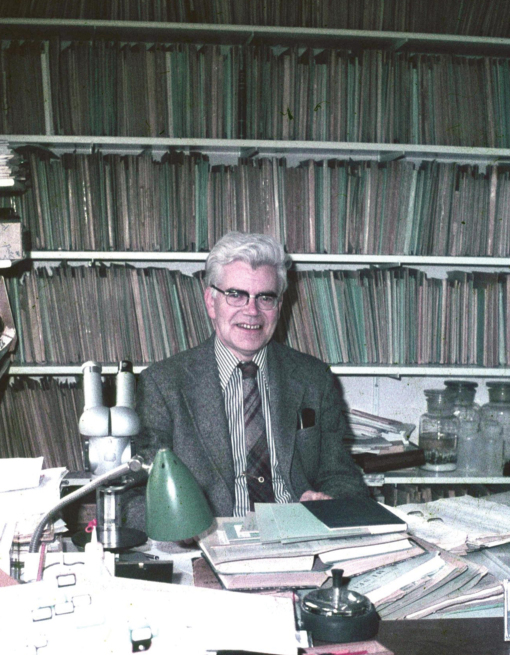
C.A.W. Jeekel at home, 1979. Image courtesy L.A. Pereira.

Up to that point in time, much of the published work on Diplopoda consisted of opportunistic descriptions of new taxa with scant attention to any form of synthesis. I was still under the malign influence of R.V. Chamberlin, an exemplar of minimal taxonomy, and my new friend in Holland was still publishing isolated descriptions, albeit with substantial amplification of his actions. However, my conversion to a different approach was catalyzed by his two 1951 papers about *Tectoporus* and *Sphaeropoeus* which showed that useful progress could be made by combining descriptions of new taxa with analysis of relevant published information. Maybe a piecemeal measure, but certainly an improvement and guide for the future work which I determined to adopt.

In 1960 I began the first of my sequence of visits to European museums searching for the types of enigmatic ancient species. After London and Tervuren, my next stop was Amsterdam, and since the KLM intown terminal was only a few minutes from the address which I had been given, I was soon looking up a steep narrow flight of stairs to the welcoming greeting and broad smile of a tall slender young man: after 11 years a Jeekel in the flesh. What ensued thereafter we later defined as a “marathon dialogue” that lasted three days and nights, fueled by many cups of coffee and punctuated by long ambulatory conversations around the city. Starved for such an exchange we exchanged views on research interests, and discussed the work of our predecessors and few contemporaries, finding that our views coincided closely on all points (admiration for Brolemann, Cook, and Pocock, for instance, despair over certain others). Cas showed me his accumulation of elegantly illustrated research projects, many of which only appeared as parts of his great *Myriapod Memoranda* series 40 years later.

He lived at the time in an archetypical bachelor two-room flat crowded with his already extensive collection of separata and books, and to my surprise, the evidence of his interest in music, particularly of New Orleans jazz: instruments: a large collection of 78 rpm recordings, and many books about prominent jazz musicians. He was an active member of a student ensemble, but even at that time music was being gradually preempted by myriapods. Regrettably we devoted little time to exchanges about personal backgrounds. I only learned that he had been born in Medan, Sumatra, and his family moved back to Holland when he was still young. But during that first visit the initiative for the *Nomenclator* was developed, and that mythical estimate of 80 000 extant species was contrived.

At the time he held a position in the Zoological Museum and was librarian for the Dutch Entomological Society. I was interested to learn to that although he passed the Rijksmuseum every day on the way to work, he had never entered that celebrated institution until he thought that his foreign visitor should be exposed to the “Night Watch” and arranged a detour to see it on one of our excursions.

Subsequent visits to Europe always included a stop-over in Amsterdam to discuss (de-brief!) the more interesting results of my delving in neglected collections and especially to follow the progress of the *Nomenclator Generum et Familiarum*. During this time, and even earlier, he had made his own pilgrimages to the museums in London, Genova, Tervuren and Geneva basically to examine material of paradoxosomatids. Curiously he never visited the great historically important collections in Munich, Berlin, and Vienna, nor endeavored to borrow material from them.

Out of a sense of filial obligation, Cas remained unmarried for many years in order to look after his aging mother. Eventually relieved of this responsibility, he encountered two major events that changed his life substantially. In early 1969 he became director of the Zoological Museum, a position assumed with much reluctance, and only because the only other likely candidate would have been “disastrous”. This new status greatly improved his standard of living, but at the expense of his research and health: the stresses of administration resulted in chronic headaches and later a painful disorder of the nerves in his face. Nonetheless he produced several papers every year during this period and completed the manuscript of the *Nomenclator*.

The second event was in every way a far happier one. In March of 1970, to use his words “I took the big step and married a lovely widow...” A. M. (Jeanne) Rijvers (known to attendees at CIM meetings as “Sjan”) was the catalyst that brought about a virtual metamorphosis in Cas’ life. I know he would not object to my sharing details of this milestone event, a remarkable and improbable story in itself, abstracted from his own account. On the bus ride to work one morning, he picked up a newspaper left behind by another passenger, and for what was surely the first time in his memory, looked at the section devoted to the promotion of new social interactions. From this unlikely departure, he selected a “woman looking for a man” entry that looked interesting and to his own astonishment, initiated the contact that proved to be the major milestone in his life. The word “destiny” might occur to the romantically inclined.

Sjan was the wife of a young school teacher in the Indies, who had moved to Holland with her two daughters following his untimely death. The axiom that “opposites attract” was never more true: it would be difficult to imagine two people so different in personalities, yet in practice so compatible. Younger, outgoing, and vivacious, she complemented and ameliorated Cas’ sedate decorum. She became a skilled collector, and accompanied him to congresses as well as excursions to Malta, North Africa, United States, and Australia. They occupied a progression of homes in Heiloo, Bergen an Zee, and Oisterwijk, each more elegant than the last, each beautifully landscaped and planted (on field trips they collected stones for the gardens as well as myriapods). Each of my European circuits during the 1970s and 1980s typically ended with a few days at the Jeekels’ home, the technical discussions conducted in such a gracious and relaxing ambience that I coined the term “The Lotus Garden” with reference to a similar experience enjoyed by Ulysses on his long return from Troy to Ithaca. That their final residence had to be in such different and constrained quarters was certainly a hardship for both. I was never able to visit during that time, perhaps as well that my last images of them would be in context of their Oisterwijk residence.

The extent to which we agreed on points of classification and nomenclature was remarkable. From the earliest days we knew that the era of random descriptive naming was over, and the direction of the future had to emphasis both janitorial work in cleaning up the inherited mess of bad taxonomy and nomenclatorial anarchy, and whatever synthesis could be accomplished with the available materials. I think the only things we disagreed on were pretty trivial (he preferred the spelling *Pollyxenus*, we parted company in several cases of generic typification, and he felt that the paradoxosomatid gonopod was generalized rather than derived). At no time was there ever an instance of verbal disharmony. We stated our opinions and ideas, to be accepted or not after reflection on their possible merits. Mentioning a visiting colleague who had brought some drawings of dubious quality, Cas reported “I did not criticize his drawings but merely put mine next to his, while explaining my ideas. . .” with the hope that such comparison might have a desired influence. He fully understood the fact that so much of taxonomy represents simply subjective evaluation of qualitative information, and was not readily amenable to being objectified. Needless to say, data matrices and cladograms were not part of his working protocols.

For me Cas Jeekel was an admired colleague with an overtone of role model and the only person who I conceded to be my master in our field of interest. His extensive research and publication, above all the monumental *Nomenclator*, set the standard for milliped systematics in the era that is now ending, and will continue to be consulted in the next one. *Ave atque vale, Magister et amicus*!

Richard L. Hoffman

Martinsville, Virginia

28 June 2011

